# VEGF, HIF-1α Expression and MVD as an Angiogenic Network in Familial Breast Cancer

**DOI:** 10.1371/journal.pone.0053070

**Published:** 2013-01-11

**Authors:** Concetta Saponaro, Andrea Malfettone, Girolamo Ranieri, Katia Danza, Giovanni Simone, Angelo Paradiso, Anita Mangia

**Affiliations:** 1 Functional Biomorphology Laboratory, National Cancer Research Centre, Istituto Tumori “Giovanni Paolo II”, Bari, Italy; 2 Unit of Interventional Radiology, National Cancer Research Centre, Istituto Tumori “Giovanni Paolo II”, Bari, Italy; 3 Molecular Genetics Laboratory, National Cancer Research Centre, Istituto Tumori “Giovanni Paolo II”, Bari, Italy; 4 Pathology Department, National Cancer Research Centre, Istituto Tumori “Giovanni Paolo II”, Bari, Italy; 5 Scientific Direction, National Cancer Research Centre, Istituto Tumori “Giovanni Paolo II”, Bari, Italy; Johns Hopkins University, United States of America

## Abstract

Angiogenesis, which plays an important role in tumor growth and progression of breast cancer, is regulated by a balance between pro- and anti-angiogenic factors. Expression of vascular endothelial growth factor (VEGF) is up-regulated during hypoxia by hypoxia-inducible factor-1α (HIF-1α). It is known that there is an interaction between HIF-1α and BRCA1 carrier cancers, but little has been reported about angiogenesis in BRCA1-2 carrier and BRCAX breast cancers. In this study, we investigated the expression of VEGF and HIF-1α and microvessel density (MVD) in 26 BRCA1-2 carriers and 58 BRCAX compared to 77 sporadic breast cancers, by immunohistochemistry. VEGF expression in BRCA1-2 carriers was higher than in BRCAX cancer tissues (p = 0.0001). Furthermore, VEGF expression was higher in both BRCA1-2 carriers and BRCAX than the sporadic group (p<0.0001). VEGF immunoreactivity was correlated with poor tumor grade (p = 0.0074), hormone receptors negativity (p = 0.0206, p = 0.0002 respectively), and MIB-1-labeling index (p = 0.0044) in familial cancers (BRCA1-2 and BRCAX). The percentage of nuclear HIF-1α expression was higher in the BRCA1-2 carriers than in BRCAX cancers (p<0.05), and in all familial than in sporadic tumor tissues (p = 0.0045). A higher MVD was observed in BRCA1-2 carrier than in BRCAX and sporadic cancer tissues (p = 0.002, p = 0.0001 respectively), and in all familial tumors than in sporadic tumors (p = 0.01). MVD was positively related to HIF-1α expression in BRCA1-2 carriers (r = 0.521, p = 0.006), and, in particular, we observed a highly significant correlation in the familial group (r = 0.421, p<0.0001). Our findings suggest that angiogenesis plays a crucial role in BRCA1-2 carrier breast cancers. Prospective studies in larger BRCA1-2 carrier series are needed to improve the best therapeutic strategies for this subgroup of breast cancer patients.

## Introduction

Breast cancer continues to be the leading cause of morbidity and mortality in European women [1]. Approximately 7% of all breast cancers present a familial breast cancer history, and around 25% of these have germline mutations in *BRCA1* and *BRCA2* genes [2]–[3]. However, family history of breast cancer remains a predictive risk factor, after carrier status for BRCA1 and/or BRCA2 mutations has been investigated. There are more of 1,500 distinct mutations, polymorphisms and variants in *BRCA1-2* genes, among which small frame shift insertions, deletions, non-sense mutations etc [4]. Both *BRCA* genes are involved in the repair of damaged DNA and both function in common pathway that is responsible for the integrity of the genome [5]. It is well known that BRCA1-2 carriers are tumors which are genetically different from sporadic cancers and they also have a different morphological phenotype [6]. Other than *BRCA1-2* genes, mutations in different autosomal dominant genes with high or moderate penetrance breast cancer susceptibility (such as *TP53*, *ATM*, *RAD50*, *PTEN*) can be also found and defined BRCAX [7]. BRCAX tumors represent a heterogeneous group of tumors whose etiology remains unclear. During the last decade new discoveries about cancer development, malignant growth and tumor progression have changed the approach to the problem of cancer. A key role is played by tumor angiogenesis, which has led to our understanding a multiplicity of biological features and subsequent therapeutic options. These in turn have improved prognosis in many cancers, including breast cancer. Angiogenesis, development of new blood vessels from preexisting vasculature, is a process which is highly regulated by a balance of pro- and anti-angiogenic molecules; the stage at which angiogenesis occurs in tumor progression is known as the “angiogenic switch” [8]–[10]. During tumor growth, angiogenesis is induced by a variety of stimuli, including pro-angiogenic growth factor, transcription factor, cell adhesion molecules and extracellular matrix proteins [11]. Among all these factors, vascular endothelial growth factor (VEGF) seems to be critical principally for blood vessel development [12]. VEGF is a glycoprotein that exerts multiple effects on tumor angiogenesis [13], [14], stimulating the formation of new blood and lymphatic vessels and increasing vascular permeability [15]. VEGF stimulates endothelial cells, mainly via its receptor VEGFR-1 (flt-1) or VEGFR-2 (flk-1). The interaction between VEGF and VEGFR-2 activates tyrosine amino acid residues contained by the intra-cytoplasmatic tail of the receptor, triggering off different signaling cascades in endothelial cells such as survival, proliferation, migration and vascular permeability [16], [17].

VEGF is also a downstream target of hypoxia inducible factor-1 alpha (HIF-1α), a transcription factor that regulates cell response to hypoxia and acts as a regulator of oxygen homeostasis [18]. Hypoxia exists in the microenvironment of many tumor entities due to structural and functional abnormality of vessels and increasing oxygen consumption caused by rapid proliferation of tumor cells. HIF-1α and hypoxia are the principle determining factors of angiogenesis and they regulate the process of invasion and metastasis, which determines the tumor aggressiveness [19]. HIF-1α is a subunit of a heterodimer, formed by HIF-1α and HIF-1β subunits. Expression levels of HIF-1α increase during hypoxia as HIF-1α is protected from ubiquitination and proteasomal degradation [20]. HIF-1α activates transcription of different genes encoding for different molecules, glucose transporters, glycolytic enzymes and VEGF [21]. Above all, HIF-1α and VEGF are major regulators of angiogenesis and of tumor progression in many types of cancer [22], [23].

New blood vessels around tumors have an important role in many tumor types, providing adequate oxygenation and nourishment. Angiogenic vessels can be visualized with immunohistochemical staining, using monoclonal antibody to endothelial cell antigens [24]. Microvessel density (MVD) has been widely investigated in different human diseases and especially in malignant tumors [11], [25], for its therapeutic potential to fight cancer.

Angiogenesis in breast cancer has been well studied. In a recent study it was demonstrated that the “angiogenic switch” occurs already at the beginning of hyperplasia, and that angiogenesis increases from the “*in situ*” to invasive tumors [13]. Moreover, Bos and collaborators have shown the association between HIF-1α and angiogenesis in invasive breast cancer [26], and the study of Hansen S. has demonstrated that MVD can independently predict poor prognosis in operable breast cancers [27]. Until now, some authors have singularly investigated the role of VEGF, HIF-1α expression or MVD in solid tumors [23], [26], [27]. Recently, Isobe and colleagues examined, for the first time, these three factors, taken together, in gastric cancer [28]. No study, to our knowledge, has before reported the role of these three markers in breast cancer and in particular in BRCA1-2 related and BRCAX cancers.

In the present study we examined VEGF and HIF-1α expression, and MVD via immunohistochemical analysis, in BRCA1-2 carriers and BRCAX cancer tissues. We also investigated whether these angiogenic markers may have a different role in the angiogenesis in BRCA1-2 carriers and BRCAX cancers compared to sporadic breast carcinomas.

## Materials and Methods

### Patients

The study group comprised 161 invasive breast cancers from 26 patients with a proven BRCA1-2 germline mutation (17 BRCA1 and 9 BRCA2), 58 BRCAX (harbored no mutations in either BRCA1 or BRCA2) and 77 sporadic patients. Information regarding the age, tumor size, nodal status, tumor grade, estrogen receptor (ER), progesterone receptor (PR), proliferative activity (MIB-1-labeling index), and HER2/neu status, provided by the Pathology Department of our Institute, and is reported in Table 1.

**Table 1:** Characteristics of BRCA1-2 (n = 26), BRCAX (n = 58) and sporadic (n = 77) breast cancers.

Variables	BRCA1-2 n (%)	BRCAX n (%)	Sporadic n (%)
**Age (median, range)**	43 (29–63)	46 (28–71)	58 (37–83)
≤median	14 (54)	30 (52)	39 (51)
>median	12 (46)	28 (48)	38 (49)
**Tumor size (cm)**			
≤2	12 (46)	27 (49)	29 (38)
>2	14 (54)	28 (51)	48 (62)
Unknown		3	
**Nodal status**			
Negative	10 (43)	18 (33)	34 (47)
Positive	13 (57)	37 (67)	39 (53)
Unknown	3	3	4
**Tumor grade**			
1		6 (11)	16 (22)
2	10 (38)	24 (43)	33 (45)
3	16 (62)	26 (46)	24 (33)
Unknown		2	4
**ER**			
Negative	14 (54)	18 (31)	18 (23)
Positive	12 (46)	40 (69)	59 (77)
**PR**			
Negative	17 (65)	20 (35)	35 (45)
Positive	9 (35)	37 (65)	42 (55)
Unknown		1	
**MIB-1**			
Negative	5 (19)	26 (46)	41 (53)
Positive	21 (81)	31 (54)	36 (47)
Unknown		1	
**Her2/neu**			
Negative	17 (65)	47 (96)	55 (87)
Positive	9 (35)	2 (4)	8 (13)
Unknown		9	14

BRCA1 and BRCA2 carrier patients were classified as having a family history after a genetic counselling program. Briefly, they were characterized according to full-length gene sequencing analyses, after a previous examination for family history of breast cancer. Familial patients were classified as having a family history of breast cancer if one of the following conditions was present: (1) at least 3 relatives (first or second degree) had breast or ovarian cancer; (2) 2 relatives younger than 50 years had breast cancer; (3) 1 relative younger than 36 years had breast cancer; (4) the patient had bilateral cancer and at least 1 relative with breast cancer (or a relative with bilateral cancer); and (5) 1 male patient had breast cancer [29], [30].

### Ethics Statement

This research as retrospective study has been approved by the Institutional Review Board. Before undergoing routine surgery, all patients signed an informed consent form authorizing the Institute to utilize their removed biological tissue for research purpose according to ethical standards.

### Immunohistochemistry

Four µm-thick sections were immunohistochemically stained using standard immunoperoxidase techniques. Slides were deparaffinized and rehydrated through a graded ethanol series and, in order to enhance antigen retrieval, the slides were then immersed in 10 mM sodium citrate buffer (pH 6.0), boiled for 30 min for VEGF, and for 45 min for CD34 and HIF-1α on a hot plate, and then allowed to cool for 20 min. Sections were incubated for 10 min in 3% hydrogen peroxide in distilled water and washed in PBS three times for 5 min. The sections were incubated overnight at 4°C with primary antibodies, washed with PBS and then incubated with biotinylated linked secondary antibodies for 60 min, and 3-amino-9-ethylcarbazole substrate-chromogen (LSAB2 System-HRP; DakoCytomation) for 15 min in the dark for VEGF and HIF-1α, and 3,3′-diaminobenzidine tetrahydrochloride (DAB, Dako Denmark) for 10 min for CD34. Counterstaining was done with haematoxylin. Known positive controls were included in each staining run. Omission of the primary antibody was used as negative controls. Immunoreactivity was assessed independently by 2 observers (A Mangia, CS), who were blinded to clinicopathological data. When a section was either uninformative or lost a case was judged as ‘not assessable’ in the statistical analysis. The rabbit polyclonal antibody anti-VEGF (A-20, 1∶150 dilution; Santa Cruz Biotechnology Inc) recognizing the N-terminus of VEGF-A of human origin was incubated overnight at 4°C. VEGF protein expression was mainly observed in the cytoplasm of tumor cells, and the immunohistochemical score (IHS) was calculated by combining the quantity score (percentage of positive stained cells) with the staining intensity score. The quantity score ranges from 0 to 4: 0 = no immunoreactivity; 1≤25% cells stained; 2 = 26–50% cells stained; 3 = 51–75% cells stained; and 4 = ≥76% cells stained. The staining intensity was scored as: 0 (negative), 1 (weak), 2 (moderate) and 3 (strong). Raw data were converted to IHS by adding the quantity score (0–4) to the staining intensity score (0–3). Theoretically, the scores can range from 0 to 7. An IHS of 6–7 was considered a strong immunoreactivity; 3–5, moderate; 1–2, weak; and 0, negative [31]. For our analyses, tumors presenting a moderate or strong score were VEGF positive (HIS:3–7). HIF-1α staining was performed with a rabbit polyclonal antibody (clone H206, dilution 1∶25; Santa Cruz Biotechnology Inc., Santa Cruz, CA, USA). The nuclear positivity of HIF-1α was defined as the presence of perinecrotic or diffuse stained nuclei. HIF-1α was regarded overexpressed when >0% of nuclei were positive according to median value cut-off (0%). Cytoplasmic staining was occasionally observed but it was not considered [26]. The HER2/neu was scored as 0, 1+, 2+ or 3+, using a monoclonal antibody (MoAb clone CB11, Novocastra Laboratories Ltd, Newcastle, UK), in accordance with the Herceptest scoring system (Food and Drug Administration accepted): 0 = no membranous immunoreactivity or <10% of cells reactive; 1+ = incomplete membranous reactivity in >10% of cells; 2+ = >10% of cells with weak to moderate complete membranous reactivity; and 3+ = strong and complete membranous reactivity in >10% of cells. Cytoplasmic immunoreactivity was ignored. Cases scoring 0 and 1+ were classified as negative, and cases scoring 3+ were classified as positive. Cases regarded as indeterminate (2+) were tested for *HER2* gene amplification by fluorescence in situ hybridization (FISH), as previously reported [30]. Briefly, using a dual probe system of different colors (PathVysion HER-2 DNA probe kit, Vysis-Olympus, Milan, Italy), the gene copy numbers of HER2 and centromeres of the corresponding chromosome 17 were retrieved. The FISH results were regarded as positive when the HER2/CEP17 ratio was ≥2.2. Cases with ratio 1.8 and 2.1 were defined as borderline. A signal was defined as significantly amplified if it was presented in approximately 20% of nuclei. Cases were considered as positive for ER (MoAb, clone 6F11, Novocastra Laboratories Ltd, Newcastle, UK), or PR (MoAb, clone PgR 636, Dako, Glostrup, Denmark) if nuclear immunoreactivity was present in >10% of tumor cells. For MIB-1-labeling index (MoAb, clone MIB-1, Dako, Glostrup, Denmark), cases were considered as positive if nuclear immunoreactivity was present in >20% of tumor cells [32].The MIB-1 cut-off represents the median value of the scores relative to all breast tumor samples analyzed during the last five years in our Institute.

### Angiogenesis assessment

Anti-CD34, a mouse monoclonal antibody (clone QBEND-10, dilution 1∶50; Novocastra Lab. Ltd, UK) was used for microvessel staining. Microvessel counting was employed for angiogenesis assessment. Immunostained tumor sections were scanned at low power magnification (40× and 100×) to identify the areas which represented the highest vascular density - so called “hot spots”. MVD was measured in three to five fields (0.75-mm^2^ per field area, with the field size measured with an ocular micrometer) with a higher density of CD34-positive cells and cell clusters at 200× magnification. The presence of a visible blood vessel lumen was not required to be defined as positive. The mean value of microvessels in three to five examined hot spots per section was then calculated. The MVD median value was used to classify each group of tumors in “high” and “low” MVD. Tissue specimens were analyzed independently by two investigators and the slides were reassessed by both investigators using a discussion microscope.

### Statistical Analysis

The two-tailed non-parametric Mann-Whitney test was used to compare the different expression levels of VEGF, HIF-1α and CD34 between different groups, and the association between VEGF, HIF-1α, CD34 and clinicopathological variables. Statistical significance was calculated for a 95% confidence interval (p<0.05). Spearman correlation from ranks was used to analyze the interaction between VEGF, HIF-1α and MVD. The results were defined as p≤0.05 for statistical significance. Calculations were performed using the Prism version 5.00 software package (GraphPad Software, San Diego, CA, USA).

## Results

### Expression patterns of VEGF, HIF-1α and MVD in breast cancer

**Figure 1:**
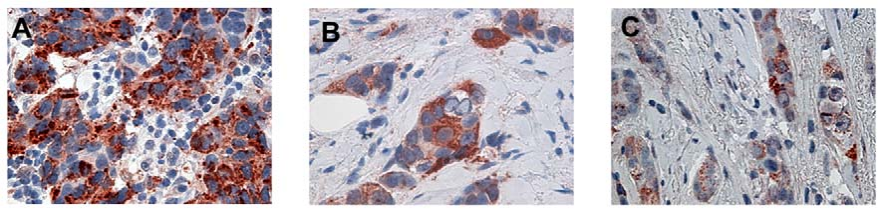
Cytoplasmic immunoreactivity of VEGF in representative tissue samples of breast cancer. (A) and (B) show VEGF overexpression in BRCA1-2 carrier and BRCAX breast cancers respectively; (C) shows low expression of VEGF in sporadic breast cancers.

**Figure 2:**
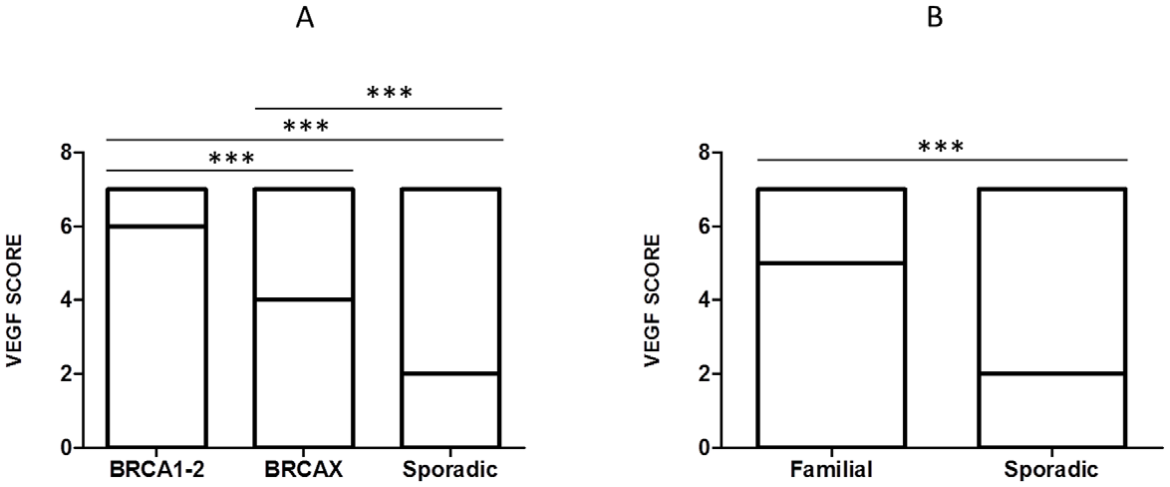
VEGF protein expression detected in different breast cancer groups. (A) VEGF expression was significantly higher in BRCA1-2 carriers than in BRCAX and sporadic group; further, VEGF expression was significantly higher in BRCAX than in sporadic cancers; (B) VEGF expression was more intensive in all familial than in sporadic cancers. *** = p≤0.0001.

**Figure 3:**
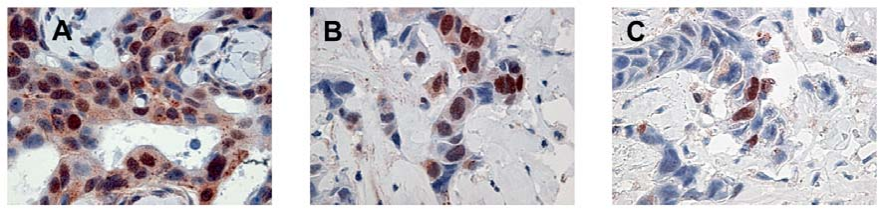
Nuclear immunoreactivity of HIF-1α in representative tissue samples of breast cancer. (A) and (B) show nuclear HIF-1α overexpression in BRCA1-2 carrier and BRCAX breast cancers respectively; (C) shows low nuclear HIF-1α expression in sporadic breast cancers.

**Figure 4:**
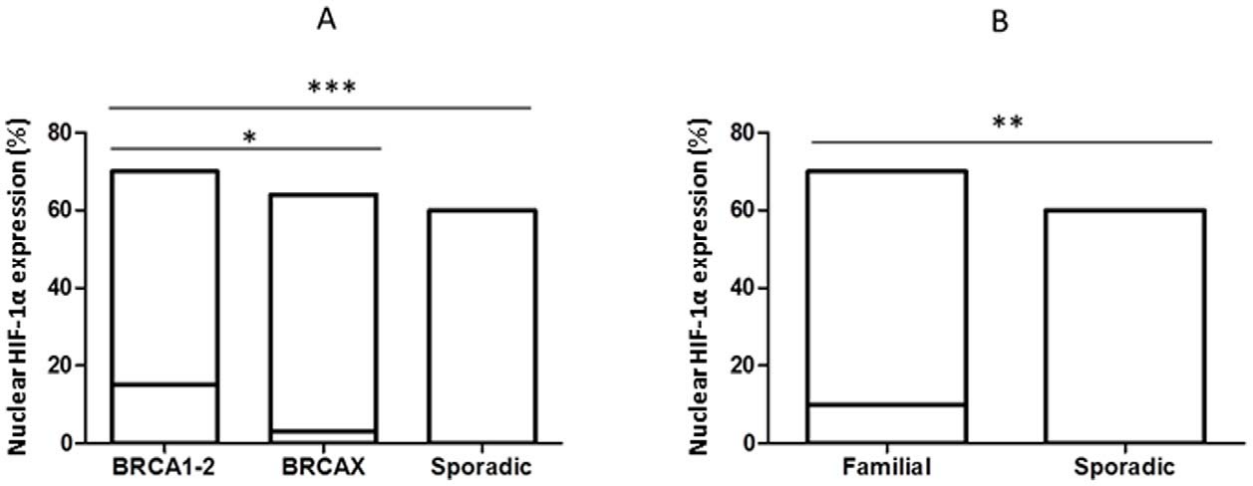
HIF-1α protein expression detected in different breast cancer groups. (A) Nuclear HIF-1α expression was significantly higher in BRCA1-2 carriers than in BRCAX and sporadic group; (B) Nuclear HIF-1α expression was more intensive in all familial than in sporadic cancers. * = p<0.05; ** = p<0.01; *** = p≤0.0001.

Cytoplasmic VEGF expression was observed in 95% (153/161) of breast cancers, and it was positive in 59% (91/153) of tumor samples. Specifically, VEGF was positive in 92% (24/26) of BRCA1-2 carriers, in 74% (42/57) of BRCAX and in 36% (25/70) of sporadic cancers. A heterogeneous intensity and expression patterns were found in the different tumor phenotypes examined. In BRCA1-2 carrier cancers the expression was homogeneous with most of the cells strongly immunoreactive for VEGF (Fig. 1A). The expression was less intensive and uniform in the BRCAX cancer tissues (Fig. 1B) and extremely weak in the sporadic cancer tissues (Fig. 1C). VEGF expression in BRCA1-2 carriers (HIS score 6) was significantly higher than in BRCAX cancers (HIS score 4) (p = 0.0001). Furthermore, VEGF expression was significantly higher in both BRCA1-2 carriers and BRCAX compared to the sporadic group (HIS score 2) (p<0.0001), (Fig. 2A). Therefore, in all familial cancers (BRCA1-2 and BRCAX) the VEGF expression was more intensive (HIS score 5) than in sporadic cancers (HIS score 2) (p<0.0001), (Fig. 2B). The nuclear staining of HIF-1α was positive in 53% (81/153) of breast cancers. Specifically, HIF-1α was positive in 85% (22/26) of BRCA1-2 carriers, in 53% (30/57) of BRCAX and in 38% (29/70) of sporadic cancers. The staining was more intensive in BRCA1-2 related cancers (Fig. 3A) than in BRCAX (Fig. 3B) and in sporadic cancers (Fig. 3C). Moreover, the percentage of HIF-1α expression was significantly higher in the BRCA1-2 carriers (median value 15%, range 0–70) than in BRCAX cancers (median value 3%, range 0–64) (p<0.05), and in the sporadic group (median value 0%, range 0–60) (p = 0.0002). No significant difference in HIF-1α expression was present between the BRCAX and the sporadic group (Fig. 4A). Furthermore, the HIF-1α expression outcome was stronger in all familial groups than in sporadic cancers (p = 0.0045), (Fig. 4B). However, Spearman analysis showed a statistically significant correlation between HIF-1α and MVD in BRCA1-2 carriers (r = 0.521, p = 0.006), and, in particular, in the familial group (r = 0.421, p<0.0001), (Table 2).

**Table 2:** Correlation between MVD and HIF-1α expression.

	MVD				
HIF-1α		BRCA1-2	BRCAX	Familial	Sporadic
	*r*	0.521	0.208	0.421	0.026
	P value	0.006	NS	<0.0001	NS

**Figure 5:**
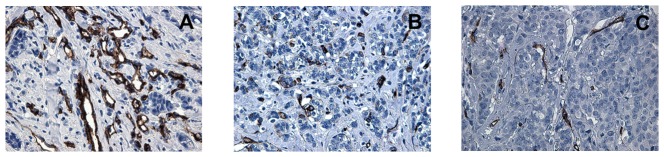
Microvessel density in representative tissue samples of breast cancer. (A) and (B) show high MVD in BRCA1-2 carrier and BRCAX breast cancers respectively; (C) shows low MVD in sporadic breast cancers.

**Figure 6:**
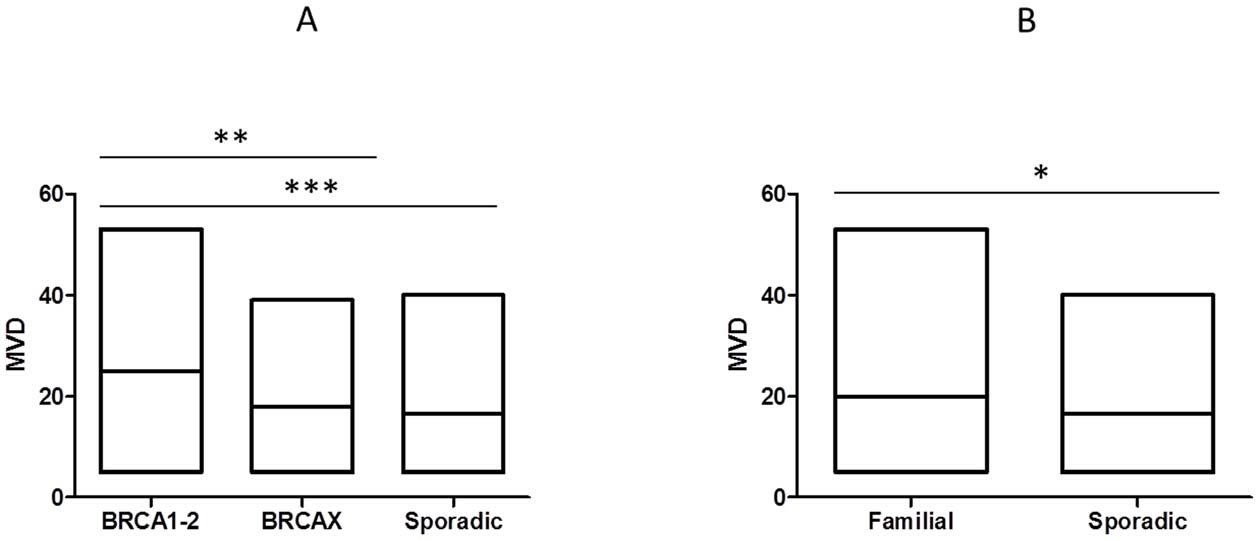
Microvessel density detected in different breast cancer groups. (A) MVD was significantly higher in BRCA1-2 carriers than in BRCAX and sporadic group; (B) MVD was more intensive in all familial than in sporadic cancers. * = p<0.05; ** = p<0.01; *** = p≤0.0001.

MVD was observed in 96% (155/161) of tumor samples examined. MVD was high in 61% (16/26) of BRCA1-2 carriers, in 51% (28/55) of BRCAX and in 53% (39/74) of sporadic cancer tissues. In BRCA1-2 carriers MVD ranged from 5 to 53, with a median of 25 microvessels/mm^2^; in BRCAX cancers MVD ranged from 5 to 39, with a median of 18 microvessels/mm2; in all familial tumors (BRCA1-2 and BRCAX) MVD ranged from 5 to 53, with a median of 20 microvessels/mm^2^, and in sporadic tumors MVD ranged from 5 to 35, with a median of 16 microvessels/mm^2^. A significantly higher MVD was observed in BRCA1-2 carrier (Fig. 5A) than in BRCAX (Fig. 5B), and in sporadic cancer tissues (Fig. 5C), (p = 0.002 and p = 0.0001 respectively), (Fig. 6A). However, the difference of MVD between BRCAX and sporadic tumors was not significant (Fig. 6A). MVD was higher in all familial than in sporadic cancers (p = 0.01), (Fig. 6B).

### Correlation among VEGF, HIF-1α expression, MVD and clinicopathologic characteristics of breast cancer

The clinicopathologic characteristics of all tumors are given in Table 1. Familiarity was significantly associated with higher tumor grade (p = 0.011) and high proliferative activity (p = 0.04) (data not shown). The correlation between VEGF expression and clinicopathologic characteristics is summarized in Table 3. When we considered all familial cancers (BRCA1-2 and BRCAX), the cytoplasmic VEGF expression was positive in 79% (66/83) of cases. In all familial cancers a higher VEGF expression was significantly associated to poor tumor grade (p = 0.0074), ER, PR negative (p = 0.0206 and p = 0.0002 respectively), and MIB-1 positive (p = 0.0044); whereas in the sporadic group VEGF positive expression was significantly associated only with ER negative status (p = 0.0296). No correlation was found in the BRCA1-2 group. Concerning HIF-1α expression no correlation was found with the clinicopathologic characteristics in BRCA1-2 and in familial groups. Only in the sporadic group there was a positive correlation with PR positive status (p = 0.0184) (data not shown). MVD was positive in 50% (42/84) of familial and in 51% (39/77) of sporadic tumors. No association between MVD and clinicopathologic characteristics in all analyzed groups was found.

**Table 3:** Correlation between VEGF expression and clinicopathologic characteristics in familial (n = 66) and sporadic (n = 25) breast cancers.

Variables	Familial*IHS Median (range)	P value	SporadicIHS Median (range)	P value
**Age (median, range)**	44 (28–71)		58 (37–83)	
≤median	5 (3–7)		4 (3–7)	
>median	5 (3–7)	NS	3.5 (3–7)	NS
**Tumor size (cm)**				
≤2	5 (3–7)		4.5 (3–7)	
>2	5 (3–7)	NS	4 (3–7)	NS
**Nodal status**				
Negative	6 (3–7)		4 (3–7)	
Positive	5 (3–7)	NS	4 (3–7)	NS
**Tumor grade**				
1–2	4 (3–7)		4 (3–7)	
3	5.5 (3–7)	0.0074	4.5 (3–7)	NS
**ER**				
Negative	5 (3–7)	0.0206	5 (3–7)	0.0296
Positive	5 (3–7)		4 (3–6)	
**PR**				
Negative	6 (3–7)	0.0002	4 (3–7)	
Positive	4 (3–7)		4 (3–7)	NS
**MIB-1**				
Negative	4 (3–7)		4 (3–7)	
Positive	5 (3–7)	0.0044	4 (3–7)	NS
**Her2/neu**				
Negative	5 (3–7)		4 (3–7)	
Positive	5 (3–7)	NS	4 (3–4)	NS

* **: BRCA1-2 + BRCAX; NS:** Not Significant.

## Discussion

In this study, we retrospectively evaluated for the first time the role of angiogenic markers such as VEGF, HIF-1α and MVD, in BRCA1-2 carrier and BRCAX breast cancers. Interestingly, we found an increase of VEGF, HIF-1α expression and MVD in BRCA1-2 carriers and BRCAX compared to the sporadic control group. Previously, other studies had found lower VEGF levels in serum from patients with BRCA1 mutations, hypothesizing a role for BRCA1 in the hypoxic response by HIF-1α stability and VEGF expression modulation [33], [34]. In our study a higher and more intensive cytoplasmic VEGF expression was evident both in tumor cells of BRCA1-2 carriers and of BRCAX cancers compared to tumor cells of sporadic cancers. The increase of VEGF expression was shown also in the BRCA1-2 carrier compared to BRCAX cancers, suggesting its central part in BRCA1-2 related carcinomas. Furthermore, we observed a higher VEGF expression in tumor cells of familial compared to those of sporadic cancers. Our results, which differ from “*in vitro*” Kang's study [34], have been confirmed by other studies which have demonstrated that the increased level of VEGF is correlated with angiogenesis and cancer development [35], showing the leading role of VEGF in tumor angiogenesis and progression in many different cancers [13], [36]–[38]. Our findings suggest a critical role of BRCA1-2 carriers in the regulation of VEGF expression and consequently the possible formation of new capillary blood vessels. It has been demonstrated in an “*in vitro*” study that the BRCA1 protein blocked VEGF promoter activity by ERα, which explains the VEGF expression increase in BRCA1 mutated cancers [39]. When VEGF was related with clinicopathologic characteristics, we found that in familial cancers VEGF expression was significantly associated with poor tumor grade and MIB-1 positive expression, confirming the close relationship with cancer progression [40]–[42]. In addition, high VEGF expression was significantly associated with ER and PR negative status in familial cancers, and with ER negative status in sporadic cancers, according to Ali, who in an “*in vitro*” and “*in vivo*” study demonstrated that a high level of oestrogen may inhibit angiogenic pathways [43].

VEGF is activated by HIF-1α during hypoxia, which is important in the progression of malignant disease and occurs in the majority of solid human tumors [44]. Hypoxic microenvironment is a feature of most tumors, and it influences many aspects of tumor biology such as angiogenesis [21] and vasculogenesis [45]. It has been demonstrated that HIF-1α is overexpressed in many human tumor types [46], [47], and its overexpression is induced by hypoxia and by oxygen-independent mechanisms. In the present study, a higher nuclear HIF-1α expression was present in BRCA1-2 carriers compared to BRCAX and sporadic cancers. The overexpression of HIF-1α in BRCA carrier cancers has been described elsewhere [7], [48]. This association confirmed the predominant involvement of HIF-1α in the development of angiogenesis in familial cancers and specifically in BRCA1-2 carrier cancers. We only found a correlation between HIF-1α expression and PR positive status in the sporadic tumors. Conversely, van der Groep showed that HIF-1α correlated negatively with the presence of ER, PR and HER2/neu [7]. The different target of patients in our series and the different antibodies used in these two studies are possible reasons for these discrepancies.

Quantification of angiogenesis, using MVD, is considered a prognostic indicator of breast cancer aggressiveness [49]. Some authors showed MVD to be an independent indicator of poor prognosis for breast cancer and inversely related with cancer survival [50], [51]. In our study, MVD was higher in BRCA1-2 carriers than in BRCAX and sporadic cancers, even if the data present in literature are controversial. Lynch indicated that tumors from patients with mutations in *BRCA1-2* showed decreased angiogenesis, compared with patients without mutations, suggesting a possible ability of BRCA1-2 carriers to escape angiogenesis [52]. In addition, we found a positive correlation between MVD and HIF-1α [28] in BRCA1-2 carrier and in all familial cancers, confirming a major aggressiveness of these tumor phenotypes. Other authors demonstrated that MVD and HIF-1α were positively associated in many types of human tumors [26], [28], [53]. We also examined the correlation between MVD and clinicopathologic characteristics, but no association was found in all analyzed groups, in agreement with other authors [54], [55]. The discrepancies in the prognostic significance of MVD can, in part be explained by considering several factors: tumor heterogeneity, pan-endothelial marker utilised (anti-CD31; anti-CD34; anti-FVIII-RA) and the variability in the choice of hot spots [56]. Heterogeneity of angiogenesis, differences in its detection methods and the variation linked to the observer did not permit MVD alone to be recognized as a reliable predictor.

In summary, we found a higher expression of VEGF, HIF-1α, and a higher MVD in BRCA1-2 carriers than in BRCAX and sporadic cancers as showed in Table 4. The angiogenesis, through the expression of VEGF, HIF-1α, and MVD plays an important role supporting the aggressive nature of BRCA1-2 carrier cancers. In this scenario, it could be hypothesized the evaluation of novel combination therapies (i.e. DNA damaging agents plus anti-angiogenic drugs) in subgroup of breast cancer patients carrying BRCA1-2 mutations. Indeed, prospective studies in larger BRCA1-2 carrier series may be warranted to improve their therapeutic opportunities.

**Table 4:** VEGF, HIF-1α expression and MVD in BRCA1-2, BRCAX and Sporadic cancers.

	BRCA1-2(N = 26)	BRCAX(N = 58)	Sporadic(N = 77)
**VEGF**	High	Moderate	Low
**HIF-1α**	High	Moderate	Low
**MVD**	High	Moderate	Moderate
